# The impact of mother's mental health, infant characteristics and war trauma on the acoustic features of infant‐directed singing

**DOI:** 10.1002/imhj.70036

**Published:** 2025-08-03

**Authors:** Raija‐Leena Punamäki, Safwat Y. Diab, Konstantinos Drosos, Samir R. Quota

**Affiliations:** ^1^ Faculty of Social Sciences Tampere University Tampere Finland; ^2^ Al Quds Open University, Gaza City, Occupied Palestinian Territories (OPT); ^3^ Nokia Research Center Espoo Finland; ^4^ Faculty of Social Sciences and Humanities Doha Institute for Graduate Studies Doha Qatar

**Keywords:** acoustic features, infant emotional responses, infant health, infant‐directed singing, maternal mental health problems, traumatic war events, Akustische Funktionen, Emotionale Reaktionen des Säuglings, Gesundheit des Säuglings, Vom Säugling gesteuertes Singen, Psychische probleme der Mutter, Traumatische Krieg Erfarenheter, Caractéristiques acoustiques, Réponses émotionnelles du nourrisson, Santé du nourrisson, Chant destiné au nourrisson, Problèmes de santé mentale maternelle, Evénements de guerre traumatisants, Características acústicas, Respuestas emocionales infantiles, Salud infantil, Canto dirigido a bebés, Problemas de salud mental materna, Eventos bélicos traumáticos

## Abstract

Infant‐directed singing (IDSi) is a natural means of dyadic communication that contributes to children's mental health by enhancing emotion expression, close relationships, exploration and learning. Therefore, it is important to learn about factors that impact the IDSi. This study modeled the mother‐ (mental health), infant‐ (emotional responses and health status) and environment (war trauma)‐related factors influencing acoustic IDSi features, such as pitch (F0) variability, amplitude and vibration and the F0 contour of shapes and movements. The participants were 236 mothers and infants from Gaza, the Occupied Palestinian Territories. The mothers reported their mental health problems, infants’ emotionality and regulation skills, and, along with pediatric checkups, illnesses and disorders, as well as traumatic war events that were also photo documented. The results showed that the mothers’ mental health problems and infants’ poor health status were associated with IDSi, characterized by narrow and lifeless amplitude and vibration, and poor health was also associated with the limited and rigid shapes and movements of F0 contours. Traumatic war events were associated with flat and narrow F0 variability and the monotonous and invariable resonance and rhythm of IDSi formants. The infants’ emotional responses did not impact IDSi. The potential of protomusical singing to help war‐affected dyads is discussed.

## 1 INTRODUCTION

Maternal infant‐directed speech (IDS), motherese and singing (IDSi) are important in serving multiple interactive, mental health and developmental functions. Their favorable acoustic features are exaggerated and soothing by nature, and protomusical IDSi encompasses (a) uniquely high, wide and smooth *fundamental pitch (F0) frequencies and F0 variation*; (b) great intensity and broad‐range *amplitude and vibration*, indicating clearness, vivacity and rich pulsation; (c) plentiful, clear and rich *formants of vocal resonance, timbre and rhythm*; (d) distinctive, diverse, contoured and multiple *F0 contour of shapes and movements of pitch variation* over time; and (e) low *tempo* and *power* (Falk & Kello, 2017; Spinelli et al., 2017; Trainor, 1996).

**Key Findings**

Practitioners can easily encourage mothers to sing with their infants since infant‐directed singing (IDSi) is a natural dyadic communication that enhances the enjoyable elements of interaction.
Our findings provide practitioners with an awareness of favorable acoustic features so that they can enhance IDSi among mothers with mental health problems and/or who have had traumatic experiences. These favorable features involve extensive variability; high, wide and vivacious vocal amplitudes and vibrations; distinct and diverse shapes and movements; and rich repertories of resonance and rhythm.
Guidelines are available for rehearsing parental singing to infants with obstetric health problems. Our findings increase knowledge of the determinants of favorable or protomusical acoustic features of IDSi and their adaptation to other vulnerable groups, such as war‐affected dyads.

**Statement of Relevance**

1. Parental singing provides multimodal and emotionally significant communication during infancy. Research on infant‐directed singing (IDSi) is scarce, but infant‐directed speech (IDS) with high vocal and acoustic quality has been found to enhance infants’ optimal emotion expression, self‐regulation and language skills, as well as healthy bonding and secure parent–infant interactions, thus contributing to infants’ mental health.
2. Newborns and infants prefer IDSi to IDS, and the former is more effective in attuning distress and evoking dyadic joy and relationship closeness.
3. Research has focused mainly on single factors that influence the acoustic quality of IDSi and IDS, and it has included only a few acoustic features. Our analysis of multiple factors (i.e., mother‐, infant‐, and environment‐related factors) that affect a comprehensive set of acoustic features of IDSi provides novel knowledge in the field of multimodal parent–infant interaction and infant mental health.

Infant mental health involves the capacity to experience, regulate and express emotions, form secure relationships, explore the environment and learn (Osofsky & Thomas, 2012; Parker, 2021). The favorable or protomusical acoustic features of IDSi enhance the preconditions of infant mental health. Mothers intuitively modify their singing to either match or alter their infants’ emotional state and arousal, which functions as training for expressing and regulating emotions (de l'Etoile, 2006; Milligan et al., 2003). IDSi, as a dyadic and reciprocal form of interaction and communication between parent and infant, activates infants’ responses and calms distress, thus functioning as training for close and secure relationships (Corbeil et al., 2016; Fancourt & Perkins, 2018; Sharman et al., 2023; Trehub et al., 2015). It sustains infants’ attention and helps children learn utterances and vocabulary, which functions as the exploration and development of language, memory and other cognitive skills (Franco et al., 2022; Saint‐Georges et al., 2013; Spinelli et al., 2017).

Due to these essential functions of IDSi, it is important to learn about the factors that increase favorable acoustic features and decrease those that are unfavorable. However, research on the determinants of acoustic features is incomplete. First, almost all studies have analyzed the determinants of IDS, but only a few have analyzed those of IDSi. Although IDS and IDSi share acoustic and vocal similarities, such as exaggeration, elevated pitch F0 and vivacious expression (Falk & Audibert, 2021; Saint‐Georges et al., 2013), protomusical IDSi differs decisively from IDS in terms of pitch patterns (melody), their temporal organization (rhythm) and amplitude and timbre (tone intensiveness). IDSi involves more prolonged vowels, syllables bound to the distinctive pitch of the melody and very high repetition and variation (Elmer, 2011). Infants are attracted to and aroused by happy sounds that convey these distinct, variable and intensive qualities (Corbeil et al., 2013; Sharman et al., 2023). They like repetition and rich rhythm, since these secure the predictability of their mothers’ messages and create a sense of mutuality (Trehub & Russo, 2020; Ullsten et al., 2018). The most decisive difference is related to the highly affective multimodality of IDSi, which is reflected in emotional, sensorimotor, symbolic, facial expressive and kinesthetic communication (Elmer, 2011; Falk & Audibert, 2021). Ample evidence confirms that infants prefer IDSi to IDS and adult‐directed singing (Saint‐Georges et al., 2013; Trainor, 1996), which may be due to the pre‐linguistic development period being especially sensitive to the communicative features of IDSi.

**FIGURE 1:**
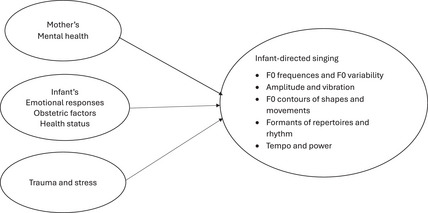
Theoretical model of mother‐, infant‐Pu and war trauma‐related factors impacting maternal infant‐directed singing (IDSi).

Second, studies have concentrated only on maternal mental health problems and, among them, on depression as a determinant of IDS. Because IDSi reflects early dyadic mutual interaction (Sharman et al., 2023), child‐related factors and wider societal contexts may have an important impact on dyadic singing communication. Therefore, this study aims to provide a more comprehensive model of the mother‐, infant‐, and society‐related factors that may impact the quality of the acoustic features of IDSi. Figure 1 presents a model of the factors that impact IDSi based on the process model of parenting by Belsky & Jaffee (2006), which in turn is based on ecological models conceptualizing the determinants of developmental phenomena as child, family and society domains (Bronfenbrenner, 1986). In this study, the participants were Palestinian mother–infant dyads living in war zones, and the scope of mental health problems was extended to include PTSD and dissociation symptoms, which are salient from threats to life and war trauma, and somatization, which is a relevant way of expressing mental pain in collective societies (Barbati et al., 2022), including ancient Middle Eastern cultures.

Third, research has provided a narrow range of acoustic features of maternal IDS by analyzing fundamental F0 frequencies, F0 variability and F0 contours (Spinelli et al., 2017). This study conceptualizes the acoustic features of IDSi more comprehensively by analyzing acoustic and vocal intensity, amplitude and vibration, formants of vocal resonance, timbre and rhythm, as well as tempo and power, in addition to fundamental F0 frequencies, F0 variation and the F0 contours of shapes and movements. These features were chosen to depict the quality of maternal singing because they reflect theoretically and empirically favorable or protomusical acoustic features of IDSi (Falk & Kello, 2017; Spinelli et al., 2017; Trainor, 1996; Ullsten et al., 2017, 2018). To conclude, this study examines how maternal mental health, infant emotional responses and health, as well as traumatic war events, are associated with a range of acoustic features of mothers singing to their 6‐month‐old infants.

### 1.1 Maternal Mental Health and Infant‐Directed Singing

We identified two studies examining the associations between mothers’ depressive symptoms and IDSi. l'Etoile and Leider's (2011) study was based on salient acoustic features of IDSi, encompassing fundamental F0 frequencies, F0 variability, starting pitch, tempo, amplitude and power, analyzed in a sufficient sample (*N =* 80). The results showed that depressed mothers’ IDSi was characterized by a higher tempo and tonality, indicating that they sang faster and louder to their infants than non‐depressed mothers. This may reflect a lack of sensitivity to the infants’ emotional states or a lack of awareness of their responses to maternal vocal communication. A study based on a subsample (*N =* 50) of the present study reported associations between maternal mental health and the vocal tone of maternal IDSi emotion expression based on judge evaluation (Punamäki et al., 2020). The results showed that depressive mothers’ singing was characterized by a high‐level angry vocal tone but that depressive symptoms did not decrease positive emotions, such as playfulness‐vivacity or love‐tenderness, or increase other negative emotions, such as fear and sadness.

Studies on IDS have shown features of flatness, slow tempo and a lack of vivacity in depressive mothers’ vocal communication. Their IDS displays especially incongruency, indicated by a lack of temporal and rhythmical modulation as well as by few responses to prompt infants’ vocalization and utterances (Bettes, 1988; Kaplan et al., 1999; Zlochower & Cohn, 1996). Bettes (1988) compared temporal parameters and F0 contours in motherese between depressed and non‐depressed mothers (self‐reported symptoms cut‐off; *N =* 36). Depressed mothers’ IDS was characterized by flatness, lacked rising, falling and U‐shaped changes of F0 contours and involved less exaggerated, differentiated and expanded intonation. Depressed mothers were also less timely and consistent in responding to their infants’ vocalization and used more unsynchronized utterances and unpredictable pauses.

Kaplan et al. (1999) found that depressed mothers’ IDS showed high flatness and low vivacity, a lack of different shape variations and fewer expanded movements of F0 contours than that of less depressed mothers (self‐reported symptoms; *N =* 20). Maternal depression was not associated with F0 variability or F0 amplitude variation in IDS, but depressed mothers’ IDS was less modulated according to their infants’ vocalization in terms of time and tempo. Finally, Zlochower & Cohn (1996) found that clinically depressed mothers’ (*N =* 35) motherese was characterized by non‐modulation and non‐synchronization, since they used longer and more inconsistent durations and latencies to respond to their infants’ vocalization than non‐depressed mothers.

### 1.2 Child Characteristics and Infant‐Directed Singing

The model in Figure 1 conceptualizes the infant‐related determinants of maternal IDSi as infants’ emotional responses, obstetric factors and health status. Research has confirmed that favorable IDSi is significant for infants’ optimal emotional development (Corbeil et al., 2016; Trehub & Russo, 2020), but no studies have suggested that infants’ emotional responses influence IDSi. The conceptualization of infants’ emotional responses as determinants of maternal singing is based on three observations. First, IDSi is decisively mutual, reciprocal, dyadic and interactive in nature, which means that infants’ emotional responses contribute to the quality of maternal singing (Sharman et al., 2023). Second, IDSi is especially salient for development during the first year of life, which co‐occurs with rigorous changes in infants’ emotional skills. At 2–6 months, infants learn to recognize, and from 7 months, they learn to express the basic emotions of fear, sadness, anger and joy (Leppänen & Nelson, 2009; Ruba & Repacholi, 2020). Approaching 18 months, they can express specific emotions and train in the more complex emotions of shame, guilt and satisfaction (Russell et al., 2003). With improved regulation skills, infants are better able to monitor, evaluate and modify their own and their caregivers’ emotional responses, which is vital for modulation and the quality of maternal IDSi. Third, infants are active partners in dyadic communication whose responses, needs, strengths and vulnerabilities influence the features of maternal IDS (Pretzer et al., 2019; Ramírez‐Esparza et al., 2017).

Despite conceptualizing IDSi as a dyadic scene in which to communicate emotions and a core function of regulation and attunement, there has been no research on the role of infants’ emotionality or regulation skills in influencing the vocal and acoustic features of maternal IDSi. However, since IDS involves mutual dyadic communication between infants and caregivers, existing studies on it may be informative. These have shown that infants’ active vocalization and utterances are likely to promote their mothers’ use of favorable IDS as well as contingency in the dyadic vocal tone of emotion expressions (Bornstein et al., 2015; Pretzer et al., 2019). Highlighting the active role of infants in dyadic exchanges, Ko et al. (2015) found evidence for matching the intensity of infants’ uttering with mothers’ speaking, as indicated by acoustic features of amplitudes and loudness. However, matching was not found in F0 frequencies or F0 variability between infants’ and mothers’ dyadic communication.

Concerning obstetric problems, there is conflicting research on the influence of preterm and full‐term birth on maternal IDS. Some studies have suggested that mothers of preterm infants show more favorable and richer linguistic and vocal features in their IDS (e.g., distinctive utterances, multiple word types and lexical variability) and aim for sensitive synchronization and balanced attunement and activation that accords with their infants’ utterances and responses (Reissland & Stephenson, 1999). On the contrary, others have found that mothers of preterm infants are at risk of over‐ and under‐stimulating their infants, since their IDS involves vocalization with high syntactic complexity, improper pauses and low levels of vocal variation (Agostini et al., 2014; Spinelli et al., 2022). Nevertheless, some studies have not found differences in the linguistic or vocal features of IDS between mothers of pre‐ and full‐term infants (Salerni et al., 2007; Suttora et al., 2020).

We identified one study on infants’ preterm birth influencing the acoustic features of maternal IDSi. A micro‐analysis of F0 contours of maternal humming to preterm infants (*N =* 36) found a predominance of sinusoidal contours, followed by bell‐shaped, rising, falling and U‐shaped, rather than linear, contours (Carvalho et al., 2022). Since the mothers used rich, varied and vivid IDSi, the results concur with findings that preterm infants invite their mothers to speak with a favorable and rich tone and melody.

We did not identify any studies on the role of infant health status in influencing the vocal, linguistic or acoustic features of maternal IDS or IDSi. Since preterm birth correlates with an increased risk for infants with illnesses and disorders (Demirci et al., 2024), it may also be that infants’ health problems can elicit mothers’ favorable and protomusical IDSi, lead to the risk of unfavorable IDSi or turn out not to be an important determinant of the acoustic features of IDSi.

### 1.3 Stress and Trauma and Infant‐Directed Singing

The participants in the present study lived in an area of war and military violence; therefore, the model in Figure 1 conceptualizes traumatic war events as potential determinants of the acoustic features of maternal IDSi that present environmental, societal and contextual factors (Bronfenbrenner, 1986). However, prior research on the impact of trauma or stress on IDS or IDSi is almost nonexistent, and we found only one study on war trauma affecting IDSi and another on parental stress impacting IDS.

A study based on a subsample (*N =* 50) of the present data found that severe war trauma regarding human losses, destruction and atrocities was associated with low levels of love and tenderness and with high levels of anger and tension in the vocal tone of maternal IDSi emotion expression. However, no associations were found between war trauma and the playfulness‐vivacity, fear or sadness of IDSi (Punamäki et al., 2020).

Spinelli et al. (2022) found that mothers’ parenting stress (problems in dyadic interactions and infant behavior and a sense of parental incompetence) was associated with less‐infant‐sensitive IDS, characterized by lower verbosity, unpredictability and higher lexical and syntactic complexity (*N =* 111). This kind of IDS is typical of adult‐directed speech and communication with older children and may thus reflect mothers’ inability to connect to infants’ needs and skills (Genovese et al., 2020).

### 1.4 Aims of the Study

This study examines how mothers’ mental health, infant emotional responses and health status, as well as traumatic war events, are associated with the acoustic features of maternal singing to their 6‐month‐old infants. Based on research on IDS and IDSi, we hypothesize that mothers’ high levels of mental health problems (depression, PTSD, dissociation and somatization symptoms) and infants’ high levels of negative emotionality and low emotion regulation skills are associated with maternal IDSi that is characterized by low and flat *fundamental F0 frequency*, low and narrow *F0 variability*, low levels of broad, vivacious and rich pulsation of *vocal amplitude and vibration*, low levels of distinctive, extensive and diverse *F0 contours of shapes and movements*, low levels of plentiful, clear and rich repertoires of *vocal resonance and rhythmicity formants* and high levels of *vocal tempo and power*. Due to the paucity of research on infant health status, trauma and stress influencing maternal IDSi (or IDS), we do not state our hypotheses but explore how infants’ health status (illnesses and disorders) and traumatic war events are associated with the acoustic features of maternal IDSi.

## 2 METHODS

### 2.1 Participants

The vocal material for this study was gathered as part of a Palestinian research project (*Protecting early development from the consequences of new weapons technology: Psychosocial, physical and family dynamic factors*). The participating mother–infant dyads lived in the Gaza Strip in the Occupied Palestinian Territory, under an international boycott and Israeli military siege since 2007 (OCHA, 2016). This study involved two assessments. At T1, the sample consisted of 502 mothers recruited at delivery in hospital maternity wards representing the four regions of the Gaza Strip (northern, central, south and Gaza City). At T2, 392 of these mothers participated from their homes when the infants were 6 months old (*M* = 6.21, *SD* = .42).

Between T1 and T2, 110 mothers (21.9%) dropped out. This was mostly because of changed home addresses and displacement due to the Israeli army shelling and bombing their neighborhoods and homes (*n* = 90), followed by the death of a baby (*n* = 13) and withdrawal for personal reasons (*n* = 7). The dropout rate was independent of infant gender, birthweight, age or education of the mother or father. However, participation at T2 was related to a longer gestation period (*t* [502] = 2.50, *p* < .01) and better newborn health (*t* [502] = 7.65, *p* < .01).

Vocal material was collected during T2 home visits by recording the mothers singing songs of their own choice to their infants. Of the 392 mothers participating at T2, 236 (60.2%) agreed to perform songs in addition to answering questionnaire interviews. The singing and non‐singing (*n* = 156, 39.8%) groups did not differ in the demographic factors of the infant gender, maternal or paternal age, number of children, father's employment and place of residence or in the obstetric and growth factors of newborn birthweight, gestational weeks and infant weight. Non‐significant *t*‐test values ranged between .086, and 1.068 and non‐significant *χ* ^2^ test values ranged between .122 and .132. However, in the singing group, fewer mothers worked or studied outside the home (7.3%) than in the non‐singing group (14.1%; *χ* ^2^ [1, 501] = 5.81, *p *= .016). In the singing group, Caesarean delivery was less common (9.9%) (17.9%; *χ* ^2^ [1, 501] = 6.12, *p* = .013) and more (67%) newborns had excellent health than in the non‐singing group (38.6%; *χ* ^2^ [4, 495] = 44.21, *p* < .004). Concerning the determinant variables of IDSi, the groups did not differ in terms of maternal mental health of depressive, total sum of PTSD, dissociation, or somatization symptoms. Non‐significant *t*‐test values ranged between .747 and 1.068, or severity of war trauma, *t* (388) = .826, *p* = .219). However, infant's positive emotionality, *t* (387) = 2.033, *p* = .021 and emotion regulation skills, *t* (387) = 1.898, *p* = .030 were at a lower level in the singing group than in the non‐singing group.

Of the 236 maternal infant‐directed songs recorded, 219 were included in the acoustic analysis. The loss of 17 songs was due to a lack of singing (speaking), poor technical quality or high noise during the recording.

### 2.2 Study procedure

In maternity wards, the midwives on duty presented the idea of this study and its prospective nature to mothers and asked them to join voluntarily between January and March 2015 (T1). The mothers willing to participate signed informed consent forms, emphasizing the participant's rights, the study's voluntary nature and the possibility of withdrawing from the study at any time without specifying the reason. The inclusion criteria were voluntariness and being pregnant in the first trimester during the 2014 war on Gaza, a 54‐day intensive military operation by the Israeli army. It involved shelling and bombing from air, land and sea and a diurnal curfew in the Gaza Strip, resulting in extensive human and material losses and displaced families (UN Human Rights Council, 2014).

Ten Palestinian fieldworkers with bachelor's degrees and previous research experience conducted home visits to interview the mothers between June and October 2015 (T2). They had previously attended comprehensive training on research tasks, ethics, interview procedures and recording mothers singing to their 6‐month‐olds. A local research team supervised the fieldwork through weekly meetings and guidance. All study procedures were conducted in Arabic. The average duration of the home visits was 90 min. The mothers received small presents for their children.

The mothers signed informed consent forms. The study procedure conformed to APA (American Psychological Association) standards on the ethical treatment of participants and complied with the Code of Ethics of the World Medical Association Declaration of Helsinki (1964–2014). Two ethical boards reviewed the study and approved its research tools and procedures (The Palestinian Health Research Council and the Helsinki Committee for Ethical Approval approved the methods, setting, and procedures of the study (PHRC/HC/126/15). Mothers who showed acute psychiatric disorders or other severe distress were guided to the local NGO clinic for consultation. Data supporting the findings are available only upon request due to privacy and ethical restrictions.

### 2.3 Measures

#### 2.3.1 Depressive symptoms

The Edinburgh Postnatal Depression Scale (EPDS; Cox et al., 1987) was applied to measure depressive symptoms at T2. The EPDS consists of 10 items that assess negative thoughts, feelings and behaviors (e.g., “I have been anxious or worried for no good reason”, “I have been so unhappy that I have had difficulty sleeping”, “I have felt sad or miserable”). The mothers responded using a four‐point scale ranging from 1 (*not at all*) to 4 (*every day*) on how often they had suffered from the described symptoms in the previous 2 weeks. A sum variable was constructed, and the Cronbach's *α* was .82, with a higher score indicating more severe symptoms. The EPDS has been found to be reliable and valid in a Palestinian context (Isosävi et al., 2020) and in another Middle Eastern context (Green et al., 2006; Montazerie et al., 2007).

#### 2.3.2 PTSD symptoms

The 16‐item scale of symptoms of post‐traumatic stress disorder (PTSD) from the Harvard Trauma Questionnaire (HTQ; Mollica et al., 1992) was applied at T2. The HTQ covers intrusive, avoidance and hypervigilance symptoms (e.g., “Recurrent thoughts or memories of the traumatic events”, “Avoiding activities that remind of traumatic event”, “Feeling jumpy and easily startled”). The mothers evaluated using a four‐point scale ranging from 0 (*not at all*) to 3 (*severely*) on how severely they had suffered from the described symptoms during the previous month. A sum variable was constructed, and the Cronbach's *α* was .91, with a higher score indicating more severe symptoms. Multiple studies have validated the HTQ among Palestinians (Salo et al., 2004; Van Heemstra et al., 2020).

#### 2.3.3 Dissociation symptoms

The 15‐item Psychological Dissociative States Scale (PDSS; Nijenhuis et al., 2003) was applied to indicate mental health at T2. The descriptions involve depersonalization and derealization (e.g., “Feeling unreal”, “Immersion in deep thought”, “Not recognizing oneself in the mirror”). The women estimated how commonly they had ever (in their lifetimes) had these kinds of experiences using a six‐point scale (1 = *never*, 6 = *all the time*). A sum variable was constructed, and the Cronbach's *α* was .77, with a higher score indicating more severe symptoms. The PDSS has been found reliable and valid among Palestinian mothers (Qouta et al., 2021).

#### 2.3.4 Somatization symptoms

Somatization was measured using the somatic symptom module of the Public Health Questionnaire (PHQ‐15; Kocalevent et al., 2013; Kroenke et al., 2002). The symptoms are based on common somatization disorders, such as headaches, faintness, dizziness and chest pain. The mothers estimated how much the described symptoms had bothered them (0 = *not at all*, 1 = *a little* or 2 = *a lot*) during the previous week. A sum variable was constructed, and the Cronbach's *α* was .80, with a higher score indicating more severe symptoms. The PHQ‐15 has not been validated on Palestinian data.

#### 2.3.5 Infant emotionality and regulation

Infant emotion expression and regulation were measured using the very short form of the Infant Behavioral Questionnaire—Revised (IBQ‐R; Putnam et al., 2014). This measure includes 37 descriptions of positive and negative infant affectivity, behavior and regulation capacity during daily situations, such as eating, bathing and separation (e.g., “When put into the bath water, how often did the baby laugh?”, “How often did the baby seem angry [crying and fussing] when you left her/him in the crib?”, “At the end of an exciting day, how often did your baby become tearful?”). Using a five‐point scale (1 = *never*, 5 = *always*), the mothers estimated approximately how often during the previous week their infant had behaved in the described way in a vignette situation. Three dimensions were identified as significant in previous research: Negative affects refer to an infant's tendency to react to stressors with anger, irritability, fear or sadness, positive affects to joy, playfulness, or pleasure, and regulation to infants abilities to be calmed when stressed (Putnam et al., 2014). Sum variables were constructed for *positive emotionality* (five items, *α* = .66), *negative emotionality* (three items, *α* = .83) and *regulation skills* (seven items, *α* = .61). The short IBQ‐R has been found to be reliable and valid (Gartstein & Rothbart, 2003), including among Palestinian dyads (Isosävi et al., 2020).

#### 2.3.6 Infant health status

The mothers reported child health at T2 by answering whether their infants were healthy (1 = healthy, 2 = illness or disability) and were provided space to report the types of illness or disability. The infants received hospital checkups, and pediatricians diagnosed non‐communicable diseases, such as congenital heart disease, developmental dysplastic hip joints, failure to thrive or rachitis.

A sum variable accounted for the values of the two overlapping indicators of infant health status.

#### 2.3.7 Home destruction

At T1, the women responded to five questions about the degree of destruction of their properties and neighborhoods during the 2014 War on Gaza—namely, whether their own houses had been bombed, the houses next door had been bombed, they were inside their homes at the time of shelling/bombardment/attack, they had found spent ammunition inside their houses and they had been displaced after the destruction (1 = *yes*, 0 = *no*). The scale was created by Qouta et al. (2021) and is valid in accurately depicting the degree of destruction and predicting exposure to warfare‐related toxic metals (Punamäki et al., 2025). A sum variable was constructed by summing the responses, with a higher score indicating more severe destruction. As an indication of validity, a researcher visited the women who reported “yes” to the first four questions and documented photographically the damage that had occurred during the military attacks on their homes and neighborhoods.

#### 2.3.8 Traumatic war events

At T2, the women responded to a 22‐item scale of traumatic war events, which covered typical experiences during Israeli military operations in the Gaza Strip (Qouta et al., 2021). Seven items asked about death and losses (e.g., death of and injury to family members, seeing a friend killed), nine about witnessing horrifying scenes (e.g., seeing deaths, explosions and bombing, experiencing threats and massacres, hearing injured people screaming) and six about life threats (e.g., fleeing for one's life due to shelling/bombing, near miss in terms of death, separation from family). The mothers reported whether they had been exposed to these events during the 2014 war on Gaza (1 = *yes*, 0 = *no*). A sum variable was constructed by summing the responses, with a higher score indicating more severe traumatic events. The scale was found to be valid in predicting trauma‐related psychiatric problems and emotional cognitive processing (Qouta et al., 2021).

#### 2.3.9 Demographic and obstetric information

The mothers reported their education (no formal, basic, college, polytechnic or university education), family employment (mother: working at home or as a professional; father: unemployed, worker or professional), family size and parental age. Midwives collected standard European and US birth register information at birth, including birthweight, length of gestation, mode of delivery, newborn health and neonatal intensive care unit.

#### 2.3.10 Acoustic features of infant‐directed singing (T2)

Fieldworkers gave the same instruction to each mother regarding singing to their 6‐month‐olds: “Many mothers sing to their babies while interacting with them. We would like to record mothers singing to their infants. Would you be willing to volunteer for us to record your singing. Please sing a song of your own choice to your infant. Please take your time. We are thankful for you letting us listen to your singing.” They recorded the singing using a Zoom H1 Handy Recorder device.

Acoustic analyses were performed using OpenSMILE (SMILE: Speech and Music Interpretation by Large‐Space Extraction; Eyben et al., 2010, 2013) software for the automatic extraction of features from audio signals and for the classification of speech and music signals. Tape‐recorded singing stimuli were digitalized at 44 kHz with a 16‐bit resolution. The software provides statistical functions (e.g., means, skewness, standard deviation and interquartile ranges [IQRs]) based on the simple moving average (SMA). The SMA is the unweighted mean of previous sequences of vocal data and can efficiently ensure that the variation and nature are aligned with the fluidity of the acoustic features of maternal singing.

Table S1 in the Supporting Information presents the acoustic features, definitions and variables (statistical functions such as means, ranges, standard deviations, SDs and IQRs). The features were as follows:

1. *fundamental F0 frequency*, indicated by mean, kurtosis, maximum, range, skewness and SD;
2. *F0 variability*, indicated by jitter (fast variation) values of difference of differences of periods (DDP) and assessed using statistical functions of mean, IQR differences between 1st and 2nd, 1st and 3rd as well as 2nd and 3rd vocal waves, kurtosis, linear regressions (linre2, linreA and linreQ), percentiles (1.0/99.0), 1st, 2nd, and 3rd quartiles, skewness and SD;
3. *vocal intensity and energy*, assessed using the root mean square (RMS) values of mean, kurtosis, maximum, minimum, range, skewness and SD;
4. *vocal amplitude and vibration*, indicated by the loudness of a signal and assessed using pulse code modulation (PCM) of discrete amplitudes by means, IQR differences between 1st and 2nd, 1st and 3rd as well as 2nd and 3rd vocal waves, kurtosis, percentiles (1.0/99.0), 1st, 2nd, and 3rd quartiles, skewness and SD;
5. *F0 contour: shape and movement*, indicated by the direction and slope of movements or F0 variations over time and assessed using F0 waves (envelopes) by separating lower envelopes and upper envelopes; the variables had the corresponding names of F0 Contour LowEnvelope and F0 Contour UpperEnvelope, both assessed by mean, IQR differences between 1st and 2nd, 1st and 3rd as well as 2nd and 3rd waves, kurtosis, percentiles (1.0/99.0), 1st, 2nd, and 3rd quartiles, skewness and SD;
6. *formants: vocal resonance and timbre*, which are the frequencies of amplified sound waves in the vocal tract, emerging as ridges or peaks in the spectrum of a voice signal; formants were assessed using mean, kurtosis, maximum, minimum, percentiles (1.0/99.0), IQR differences between 1st, 2nd, and 3rd quartiles, quartiles 1, 2, and 3, skewness and SD;
7. *rhythmicity throu*gh bandwidths, referring to the duration, stress, quality and repetition of sounds and indicated by bandwidth (a voice signal expressing the difference between the higher/upper and lower frequencies) and assessed using mean, kurtosis, maximum, minimum, percentiles (1.0/99.0), IQR differences between 1st and 2nd, 1st and 3rd as well as 2nd and 3rd waves, 1st, 2nd, and 3rd quartiles, skewness and SD; and
8. *vocal tempo and power*, indicated by attack time, which refers to the initial impulse required to create oscillation, tempo in vocal utterances or a rise in amplitude over time, and assessed by mean, IQR differences between 1st and 2nd, 1st and 3rd as well as 2nd and 3rd waves, kurtosis, maximum, minimum, percentiles (1.0/99.0), quartiles 1, 2 and 3, range, skewness and SD of time.

#### 2.3.11 Translation

All measures were in Arabic. The following scales of maternal mental health (i.e., HTQ EPDS, and PDSS‐15) and infant emotional responses (IBQ‐R) were available in Arabic, having been translated and validated in earlier studies (Green et al., 2006; Isosävi et al., 2020). One bilingual researcher translated the PHQ‐15 somatization scale from English into Arabic, and another conducted a back translation to check for accuracy.

### 2.4 Data analysis

We used IBM SPSS‐27 statistics to examine demographic distributions and conducted factor analyses of the variables of acoustic features. The OpenSMILE acoustic feature extractor provided a large number of variables or statistical functions (see Table S1). To use structural equation modeling (SEM) with efficient power, we had to reduce the number of observed and potential latent variables to be acceptable for modeling the available data (*N* = 219). We used a freely available calculator of a priori sample sizes for SEM (Soper, 2022) to test whether the data from 219 mothers were sufficient. This calculation was based on a statistical power of Cohen's *d = *.80, an anticipated effect size of .30 and a level of significance of *α* = .05 (Wolf et al., 2013). The calculation, with six latent variables (one for maternal mental health, one for infant emotional responses and four acoustic features) consisting of 33 observed variables (four for maternal mental health, three for infant responses and 26 for acoustic features) and three additional sum variables (infant health status, traumatic war events and home destruction), resulted in a recommended minimum sample size of 200. The statistically calculated sample size was larger than the rule of thumb, suggesting a SEM sample size of five cases for each observed or sum variable (Nunally, 1978). In our model, there were 36 variables, thus amounting to 180 variables.

We proceeded with three steps to reduce the number of acoustic feature variables (statistical functions) and to detect their conceptually unified dimensions. First, we omitted variables that did not show multivariate normality based on graphical information from probability–probability plots and histograms with normality curves. Second, we conducted an exploratory factor analysis with varimax rotation on the remaining acoustic feature variables. The criteria for omitting variables were a small communality value (<.50) explained by the factor, low and unstable factor loadings (<.40) and loadings on two different factors (Hair et al., 2006). Third, the measurement models of the SEM further detected variables with nonsignificant standardized regressions to be omitted.

To test the research hypotheses and questions of factors impacting maternal IDSi, we applied SEM (AMOS 26.0; Arbuckle, 2011). The model included five independent variables: two latent variables (mothers’ mental health and infants’ emotional responses) and three sum variables (infant health status, house destruction and traumatic war events). The dependent variables were four latent variables of acoustic features of maternal IDSi (F0 variability, vocal amplitude and vibration, resonance and rhythm: formants and bandwidths and shapes and movements: F0 contours) (see Constructing Acoustic Variables).

SEM with latent, observed and sum variables was chosen for the following reasons. First, it provided tools to analyze the measurement models of highly correlating observed variables of acoustic features (e.g., statistical functions of means, minimum, maximum, range, kurtosis and SD that typically correlate), thus avoiding the risk of multicollinearity with large measurement errors. Second, it allowed us to conceptualize acoustic features as latent variables, which is novel in voice analyses, and helped in aggregating observed acoustic feature variables in a model representing the core characteristics of IDSi. Latent variables are superior to sum variables because contributing observed variables have their own unique weights, and measurement error is controlled to guarantee full reliability (Fan et al., 2016). Third, it enabled the simultaneous assessment of multiple associations between mother‐, infant‐, and war‐related determinants and acoustic features, making it superior to regression models that would test their impacts on separate acoustic features.

The criteria for model fitness were the approximate comparative fit index (CFI > .90; a measure of relative model fit), the Tucker–Lewis index (TLI > .90; a measure of a non‐normed fit index), the RMS error of approximation (RMSEA < .06; indicating parsimony of the model) and *χ* ^2^/df (values 1–3 measured in the absolute model fit) (Brosseau‐Liard & Savalei, 2014; Brosseau‐Liard et al., 2012). The significance level was defined as a *p*‐value of less than .050 for *β* values in the measurement models and SEM associations.

## 3 RESULTS

### 3.1 Descriptive statistics

Table 1 presents the demographic, obstetric and health factors of the mothers and infants. The average age of the mothers was 26 years (*SD* = 6.02). A quarter had basic education, a third had high school or college education, and another third had polytechnic education. Of the mothers, 2% had no formal education. The majority of the mothers worked at home (i.e., outside working life; 93%), and the rest worked as blue‐collar workers (e.g., teachers and nurses). Of the fathers, 41% were workers and a third were blue‐collar employees, while approximately a quarter were unemployed. Almost half of the families lived in urban areas, and a third lived in refugee camps. Gaza City and the northern, central and southern regions of the Gaza Strip were equally represented in the subsample of singing mothers.

**TABLE 1:** Demographic, obstetric and health characteristics at birth (T1) and 6 months (T2).

	Participants^a^	
	%	n
Mother's age (years)		
16–24	41.6	91
25–29	30.6	67
30–34	17.4	38
35–39	8.2	18
40–44	2.3	5
Number of children		
First child	30.6	67
1–3	50.2	110
4–6	15.5	34
7–10	3.6	8
Mother education		
No formal education	2.1	4
Basic education	25.0	55
Gymnasium/College	34.4	76
Polytechnic	32.1	70
University	6.4	14
Mother employment		
Works at home	92.7	203
Works in profession	5.9	13
Student	1.4	6
Father employment^b^		
Unemployed	23.6	51
Worker	41.2	89
Blue collar	32.4	70
High professional	2.8	6
Region of the Gaza Strip		
North	15.5	34
Middle	34.7	76
South	28.8	63
Gaza City	21.0	46
Type of residence		
Urban area	45.9	100
Village	21.6	47
Refugee camp	32.6	71
Child's sex		
Girl	53.4	117
Boy	46.6	102
Gestational age (weeks)		
< 37	3.4	7
37–40	96.6	201
Birth weight (gr)		
<2500	3.4	7
2500–3499	55.2	112
3500–4499	39.9	81
>4500	1.5	3
Newborn health		
Excellent	67.4	145
Good	31.6	68
Health problems^c^	1.0	2
Birth defect		
ICD 10 diagnosis	4.6	10
Not defect	95.4	209
Type of delivery		
Normal vaginal	82.6	180
Caesarean	17.4	38
Infant health at 6 months (mother‐reported)		
Healthy	82.8	181
Illness or disability	17.2	37
Infant health (pediatric diagnosis)^d^		
Not diagnosis	86.3	170
Non‐communicative disease	13.7	27

^a^ *N* = 219; Numbers differed due to missing data.

^b^ Workers are e.g., building, factory, traffic and agriculture worker, blue collars are e.g., teacher or officer, high professionals are e.g., doctor or engineer.

^c^ Combines reasonable and severe problems (both *n* = 1).

^d^ The diagnoses were e.g., CHD, congenital heart disease; ASD, atrial septal defect; VSD, ventricular septal defect; DDH, developmental dysplastic hip joint; FTT, failure to thrive, asthma, and rachitis (WHO, 2009).

Approximately a third of the mothers had their first child, while half had previously had 1–3 children. The sample included slightly more girls than boys. Regarding obstetric data, the average duration of gestation was 39.23 weeks (*SD* = 1.77) and the average birthweight was 3348.38 grams (*SD* = 527.96). The prevalence of preterm delivery (<37 weeks) was 3%, that of low birth weight (<2500 grams) was 3% and that of birth defects was 5%. The majority had had vaginal normative deliveries. Of the newborns, 5% had a birth defect, such as congenital heart defects or club foot. At T2, when the infants were 6 months old, the mothers reported that 17% suffered from illnesses or disorders, such as neurological disease, respiratory illness, bone weakness or heart problems. In the pediatric checkups, 14% of the infants were diagnosed with noncommunicable disease, such as congenital heart disease, developmental dysplastic hip joint, failure to thrive or rachitis.

### 3.2 Constructing acoustic variables

Preparation for the SEM analysis involved data reduction and constructing the observed composite variables. First, Table S1 in the Supporting Information presents the names of the acoustic features, their descriptions and the corresponding single variables with their original names (i.e., statistical functions of acoustic features, such as jitter, loudness, F0 contours, formants or bandwidths). The variables that did not show multivariate normality variance were omitted from further analyses, and their names are reported in notes below Table S1 in the Supporting Information. Only in the F0 contours: vocal shapes and movements did all the variables satisfy the multivariate normality criteria, while in other acoustic features, 1–7 single variables were non‐normal and thus omitted.

Second, Table S2 in the Supporting Information presents the results of factor analysis conducted to detect the conceptually salient dimensions of the acoustic feature variables and to further reduce the number of single variables (statistical functions). The exploratory factor analysis resulted in five factors that explained 66.51% of the variation. Before naming the dimensions, several variables were omitted due to low communality, low factor loading or double loading (on two factors). The results show that all the variables featuring fundamental F0 frequencies and vocal intensity and energy had to be omitted according to these criteria; consequently, factor V was not valid. The contents of the factors or acoustic feature dimensions that emerged were as follows: *I F0 variability*, covering four jitterDDP variables and one F0 contour variable (upper envelope skewness); *II vocal amplitude and vibration*, covering six PCM loudness variables; *III resonance and rhythm*: *formants and bandwidth*, covering three formant variables and five bandwidth variables; and *IV vocal shapes and movement: F0 contours*, covering four F0 contour lower‐envelope and three upper‐envelope variables.

### 3.3 Measurement models for mental health, infant emotional responses and acoustic features

Table 2 presents the measurement models of the observed variables of the latent variables of maternal mental health and infant emotional responses and the four latent acoustic variables of IDSi. The observed variables of maternal mental health loaded statistically significantly on the corresponding latent variables, whereas the three observed variables of infant emotional responses were non‐significant. All observed variables of the acoustic features of IDSi loaded statistically significantly on the four corresponding latent variables of F0 variability, vocal amplitude and vibration, resonance and rhythmicity: formants and bandwidth and shapes and movements: F0 Contours.

**TABLE 2:** Loading estimates of observed variables of maternal mental health, infant emotional responses and acoustic features of maternal infant‐directed singing: Statistical functions for corresponding latent constructs of the structural equation model (SEM).

Observed variables for latent constructs	Unstd *β* ^a^	SE	Std *β*	*t*‐tests^b^
*Maternal mental health*				
PTSD symptoms	1.000		.677	
Depressive symptoms	1.070	.112	.755	9.538^****^
Dissociation symptoms	1.714	.174	.840	9.845^****^
Somatization symptoms	1.220	.136	.388	5.279^****^
*Infant emotional responses*				
Positive emotionality	1.00		.346	
Negative emotionality	−.008	.056	−.219	−.136
Regulation skills	.028	.199	.145	.140
Maternal infant‐directed singing				
*F0 variability*				
F0finEnv_Skewness	1.000		−.637	
JitterDDP LinregerrQ	.017	.001	.911	16.319^****^
JitterDDP Linregc2	.046	.004	.775	13.213^****^
JitterDDP Skewness	−1.569	.125	−.806	−12.553^****^
JitterDDP_Standard deviation	.053	.003	.976	17.029^****^
*Vocal amplitude and vibration*				
PCM loudness Skewness	1.000		−.708	
PCM loudness IQR 1–2	.195	.018	.793	10.651^****^
PCM loudness IQR 2–3	.194	.017	.844	11.145^****^
PCM loudness Percentile 1.0	.106	.012	.649	9.175^****^
PCM loudness Percentile 99.0	.175	.124	.689	9.472^****^
PCM loudness Quartile_1	.320	.031	.779	10.412^****^
*Resonance and rhythm: Formants and bandwidths*				
Formant_0 Mean	1.000		.536	
Formant_0 Quartile_2	6.918	.553	.887	12.498^****^
Formant_0 Skewness	−.003	.000	−.632	−7.833^****^
Bandwidth_0 Kurtosis	.101	.012	.792	8.242^****^
Bandwidth_0 Quartile_2	6.750	.719	.997	9.385^****^
Bandwidth_0 Quartile_1‐2	2.958	.303	.980	9.778^****^
Bandwidth_0 Quartile_3	6.363	.678	.997	9.384^****^
Bandwidth_0 Skewness	−.012	.001	−.912	−9.031^****^
*Shapes and movements: F0 contours*				
F0final IQR 2–3	1.000		.338	
F0finEnv IQR 1–2^c^	1.216	.236	.609	5.150^***^
F0finEnv IQR 2–3	1.003	.116	.459	8.464^****^
F0finEnv Kurtosis	−.277	.050	−.632	−5.517^****^
F0final IQR 1–2^c^	1.020	.222	.609	4.595^****^
F0final Kurtosis	−.321	.059	−.779	−5.469^****^
F0final Percentile 99.0	.919	.849	.519	4.962^****^

*Notes*: The values of 1 of the unstandardized regression estimates (Unstd *β*) refer to parameters fixed to the measurement models.

^b^ The model fitness indices are interpreted concerning the total model including latent variable measurement models and predicting coefficients, reported in Table 3.

^c^ F0finEnv‐variable name refers to lower envelopes (sound waves) and F0final‐variable name to upper envelopes.

*** *p* < .001,

**** *p* < .0001; *N* = 219.

All observed variables had estimation loadings of *β* > .50, except for somatization symptoms (*β*  = .39) on the maternal mental health latent construct and F0final IQR 2–3 (*β*  = .34) and F0fin IQR 2–3 (*β*  = .46) on the shapes and movements: F0 contours latent construct. These observed variables were kept in the models for theoretical and statistical reasons. The measurement models were part of the SEM, and the fit indices of the final model with latent variables and sum variables are presented in Table 3.

### 3.4 Mother, infant and war factors impacting the acoustic features of IDSi

**FIGURE 2:**
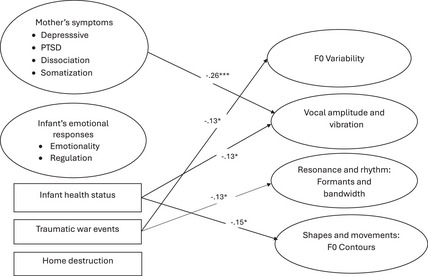
Structural equation model of the impact of mother's mental health, infant characteristics and war trauma on maternal infant‐directed singing (IDSi).

Figure 2 illustrates the SEM results of the tested model of factors impacting acoustic features of maternal IDSi, and Table 3 summarizes the non‐standardized (Unstd *β*) and standardized (Std *β*) estimates, standard errors, *t*‐tests, explained variance (percentages based on *R* ^2^) and fit indices. The fit indices showed a moderate fit of the SEM model with the data (CFI = .90, TLI = .90, RMSEA = .086 [90% CI: .080–.091], *χ* ^2^/df = 2.61).

**TABLE 3:** Structural equation model (SEM) on mother‐related, infant‐related and war trauma determinants of maternal infant‐directed singing (IDSi) involving independent variables of latent constructs of mother mental health and infant emotional responses and manifest construct of infant health, and dependent variables of latent constructs of acoustic features of IDSi: parameter estimates (coefficients) associations and model fit indices.^a^

	Maternal infant‐directed singing							
	F0 variability				Vocal amplitude and vibration			
Determinants of MDSi	Unstd *β*	SE	Std *β*	*t*‐tests	Unstd *β*	SE	Std *β*	*t*‐tests
Mother's mental health	−.125	.148	−.056	−.845	−.336	.087	−.260	−3.870^***^
Infant's emotional responses	−.012	.088	−.039	−.137	−.002	.018	−.013	−.136
Infant health problems	−.071	.160	−.027	−.445	−.189	.086	−.133	−2.210^*^
Home destruction	−.018	.037	−.031	−.496	−.017	.020	−.049	−.856
Traumatic war events	−.025.	.012	−.127	−2.001^*^	−.001	.006	−.008	−.133
Explained variance	2%				9%			

	Maternal infant directed singing							
	Resonance and rhythm: Formants and bandwidths				Shapes and movements: F0 contours			
Determinants of MDSi	Unstd *β*	SE	Std *β*	*t*‐tests	Unstd *β*	SE	Std *β*	*t*‐tests
Mother's mental health	−.140	.674	−.015	−.207	−.984	.225	−.027	−.437
Infant's emotional responses	.001	.022	.001	.027	−.153	.120	−.031	−.137
Infant health problems	−.438	.731	−.039	−.599	−.824	.586	−.130	−1.965^*^
Home destruction	−.111	.170	−.044	−.651	−.427	.570	−.045	−.749
Traumatic war events	−.127	.058	−.154	−2.207^*^	−.124	.188	−.039	−.656
Explained variance	2%				2%			

*Notes*: Model fit indices: *χ* ^2^ = 1,436,983, df = 551. *p* = .0001; *χ* ^2^/df = 2608, CFI = .90; TLI = .90; RMSEA = .086 [90% CI: .080–.091]. *N* = 219.

^a^ The fit indices are from total SEM models with the measurement models of latent constructs on maternal mental health, infants’ emotional responses and acoustic features of IDSi in Table 2.

* *p *< .05;

*** *p* < .001.

The results showed, as hypothesized, that severe maternal mental health problems were significantly associated with a low level of breadth, vivacity and rich pulsation of vocal amplitude and vibration (*β* = −.26, *p* = .001) in IDSi. However, contrary to the hypothesis, maternal mental health did not have significant associations with F0 variability, resonance and rhythm: formants and bandwidth or shapes and movements: F0 contours of IDSi. Against our hypothesis, infants’ emotional responses of negative emotionality and dysfunctional regulation were not significantly associated with any of the acoustic features of maternal IDSi.

The results further showed that infants’ health problems were associated with low levels of breath, vivacity and rich pulsation of vocal amplitude and vibration (*β* = −.13, *p* = .027) and distinctive, extensive and diverse vocal shapes and movement: F0 contours (*β* = −.13, *p *= .050). Severe traumatic war events were associated with low and narrow F0 variability (*β* = −.13, *p* = .045) and with a low level of plentiful, clear and rich resonance and rhythmicity: formants and bandwidth (*β* = −.15, *p *= .027). Home destruction was not significantly associated with any of the acoustic features of maternal IDSi.

## 4 DISCUSSION

This study analyzed salient mother‐, infant‐, and war trauma‐related factors impacting the acoustic features of IDSi. The hypotheses were substantiated concerning maternal mental health, infant health and traumatic war events, all contributing to unfavorable or non‐protomusical maternal singing. Mothers’ mental health problems, such as depression and PTSD, and infants’ health problems, such as neurological and heart disorders, were associated with maternal IDSi characterized by flat, inarticulate, even and rigid pulsation, beat and tone of vocal amplitude and vibration. Infant health problems were also associated with unclear, restricted and scant shapes and movements of F0 contours in maternal IDSi. Severe traumatic war experiences, such as death and destruction, were associated with maternal IDSi characterized by low and narrow F0 variability and homogenous tone and scarce, lifeless and monotonous vocal resonance, timbre and rhythm of formants and bandwidths. Infants’ emotionality and emotion regulation were not associated with the acoustic features of maternal IDSi.

The fitness indices of the tested SEM model were reasonable, but the explained variation was very low. Therefore, the findings must be interpreted with caution and should be replicated in more multicultural mother–infant interactions.

### 4.1 Poor mental health risks vivacity and melody of singing

Severe maternal mental health problems significantly hampered only one acoustic feature dimension: vocal amplitude and vibration. The singing of mothers suffering from depressive, PTSD, dissociation or somatization symptoms lacked richness, vivacity, articulation, dynamic lilting and a melodious tone. The reason why mental health problems did not harm the quality of other acoustic features, such as F0 variance, resonance and rhythm of formants or shapes and movements of the F0 contour, may relate to the degree of intentionality, control and conscious access in regulating vocal expressions and to the salience of matching 6‐month‐old infants’ and mothers’ vocal communication.

Singing involves complex tripartite vocal motor and somatosensory control processes. The lowest levels of control and conscious access involve reticular motoneurons, followed by initiative and emotionally motivated vocal responses. The highest levels of control and conscious access involve learned and modulated vocalizations of speaking and singing (Jürgens, 2009; Zarate, 2013). We can speculate that vocal amplitude and vibration, reflecting intensity, clearness and energy of vocal extension, pulsation and oscillations, may be the most difficult aspects to consciously regulate and control, whereas variations, rhythm and movements in singing can be more easily learned and thus controlled.

Earlier research has shown that, in addition to flatness, lack of vivacity, slow tempo and low energy, a characteristic of the IDS of depressive mothers is incongruency—that is, a lack of temporal and rhythmical modulation and few prompting responses to infants’ vocalizations and utterances (Bettes, 1988; Kaplan et al., 1999). Our results specify that mothers suffering from mental health problems face difficulties in modulating and controlling the vocal amplitude and vibration of their singing so that it is nuancedly matched with the infants’ responses. The function of soothing infants’ distress is especially crucial in the early months, and maternal IDSi is particularly effective due to the appropriate oscillation and breadth of beat and tempo (Corbeil et al., 2016). Mothers with mental health problems are often incapable of recognizing and congruently responding to their infants’ needs (Slomian et al., 2019) and, in particular, may fail to match the intensity and power of stimulation with their infants’ emotional states and developmental skills. Subsequently, infants can feel overwhelmed due to hyperstimulation or feel neglected because of low or absent stimulation, in turn signifying difficulties in their self‐regulation (Field et al., 2006).

Understanding the role of acoustic and vocal modulation in dyadic interactions is valuable. The finding that maternal mental health problems were especially harmful to the vocal amplitude and vibration of IDSi suggests that therapeutic interventions are required that increase mothers’ awareness of their less controlled and unconscious ways of stimulating and communicating with their infants. Effective elements of music‐based therapies that enhance optimal infant–mother interaction involve the accurate recognition and rich vocal and acoustic expression of emotions, multisensorial stimulations of touch, movement, and voice and vivacious and rich auditory bonding (Ullsten et al., 2017, 2018; Trehub & Russo, 2020).

Considering future research, in addition to comprehensive analyses of different acoustic features, it is necessary to assess the degree of how well mothers modulate their IDSi by harmonizing, attuning and fitting the singing with their infants’ responses. Optimally, mothers even accommodate the intensity, energy, vocal extension, pulsation and oscillation tempo of their IDSi to follow their infants’ breathing and attuning rhythm (Trainor, 1996), reflecting interactive synchronization and maternal receptiveness to their infants’ emotions and needs (Ramírez‐Esparza et al., 2017; Trehub & Russo, 2020).

Our study conceptualized maternal mental health more comprehensively than earlier studies that focused only on depressive symptoms as determinants of IDS. It would be informative to identify the specific dynamics of different mental health problems in relation to the acoustic features of maternal IDSi. Different dimensions of PTSD and dissociation symptoms may have unique impacts on IDSi. Mothers suffering from intrusive and hyper‐arousal symptoms and dissociations due to PTSD may show agitated and unpredictable rhythmic, vocal and tactile interactions with their infants, since traumatic memories involuntarily intrude into their minds and also make them highly vigilant to threatening cues in a safe environment, which detaches their minds from those of their infants (Enlow et al., 2014; Isosävi et al., 2020). Similarly to depressive symptoms, avoiding PTSD symptoms may, in turn, make mothers withdrawn and emotionally distant, which can be reflected in their IDSi as flat, lifeless and with a narrow tempo, amplitude and vibration.

### 4.2 Infant health problems hamper the vivacity and diversity of singing

Infants’ health problems, including neurological, respiratory and heart disorders, were associated with two kinds of unfavorable or non‐protomusical acoustic features of IDSi. Maternal singing was characterized by a flat, inarticulate, even and rigid pulsation, beat and tone of vocal amplitude and vibration and by the unclear, restricted, tedious and meagre shapes and movements of F0 contours. The results concur with findings that infants’ obstetric problems are associated with maternal IDS involving low levels of vocal variation and improper syntactic complexity and pauses (Agostini et al., 2014; Spinelli et al., 2022). In other words, our results do not support the idea that mothers improve the acoustic quality of their vocal communication when infants are weak, fragile or at developmental risk by, for instance, intensifying, widening and enriching their singing (Carvalho et al., 2022).

Mothers of infants suffering from illnesses or disorders may feel deeply worried, exhausted and helpless, which can negatively impact the quality of their IDSi. Infants who have health problems may be less responsive during dyadic communication and show dissatisfaction and withdrawal during singing interchanges. Subsequently, mothers can become tired of trying to coax their infants into participating and gradually and often unconsciously decline the amplitude and vibration and constrict and blunt shapes and movements of their singing.

IDSi is a scene of the dance steps of reciprocity represented in mutual dyadic vocal communication, interaction and synchrony (Feldman, 2007; Stern, 2014). Therefore, it is not easy to explain why we failed to find any impact of infants’ emotionality and emotion regulation on maternal IDSi. In addition, considering the importance of IDSi in soothing, calming down and consoling distressed infants (Lense et al., 2022; Trehub & Russo, 2020), it is surprising that the valence of infants’ emotionality and regulation skills did not influence IDSi. The danger of the dyadic environment and methodological deficits may explain why neither infants’ positive and negative emotionality nor ways of regulating emotions contributed to IDSi.

The Palestinian mothers sang to their infants under conditions of military siege during the aftermath of a massive Israeli military operation and under constant threat of war. Studies from the Middle East have illustrated that life threats and war atrocities greatly shape mother–infant dyadic interaction. Mothers feel guilty about not being able to protect their children, devote themselves to overprotection, are vigilant to dangers and express feelings of worry, fear and helplessness (Isosävi et al., 2020; Kaitz et al., 2009). It is possible that the mothers were immersed in their own worries about the survival of their infants, which resulted in them disregarding the infants’ nuanced emotional messages. Thus, mothers’ difficulties in recognizing their infants’ emotional states of mind and regulation attempts may explain why these did not have an impact on the acoustic features of their IDSi.

Considering the methodological explanation, the measurement model of the three dimensions of infant emotional responses was unsuccessful. However, due to the sample size/variables ratio, we could not use positive and negative emotionality and regulation as separate observed variables in the model. This methodological deficit may explain the null results.

Developmental issues cannot explain the non‐significance of infants’ emotionality and emotion regulation in IDSi, since 6‐month‐olds are increasingly skilled at emotion recognition, expression and regulation, as well as active in eliciting mothers’ emotions and inviting reciprocal communication (Ruba & Repacholi, 2020; Leppänen & Nelson, 2009). Maternal IDSi conveys a variety of emotions, and thus, optimal singing facilitates infants’ emotional development (Corbeil et al., 2016; Lense et al., 2022).

### 4.3 War trauma interferes with the singing repertoire and rhythmic richness

Traumatic war events were associated with unfavorable acoustic features of maternal IDSi. The singing of mothers exposed to human and material losses, atrocities and life‐threats was characterized by poor, low and narrow F0 variability, a limited repertoire of resonance and a lack of rhythmic richness of formants. These features precisely oppose the acoustic speech characteristics that reflect positive emotions and happiness (Juslin & Laukka, 2003). Our findings on IDSi concur with studies showing that traumatic events in particular interfere with joy, playfulness and a positive atmosphere in mother–infant interaction (Dozio et al., 2020; Lachman et al., 2019; Isosävi et al., 2020).

There is evidence that favorable and protomusic acoustic features in the infant–mother vocal communication of IDS and IDSi can pivotally enhance infants’ emotional well‐being and mental health (Spinelli et al., 2017; Ullsten et al., 2018; Punamäki et al., 2024).Traumatic war events thus cause a vicious circle for child development, impeding precisely the natural endeavors that could protect children in war zones. Among the beneficial resources is mothers’ singing, characterized by high, wide and smooth variation and distinctive and wealthy shapes and movements.

International organizations and human rights activists have gone to great effort to protect infants, children and maternity care during wars. However, technically sophisticated modern warfare is targeted especially at civilians, and war traumatization presents severe risks for child development and mental health (Hazer & Gredebäck, 2023). Infants’ and toddlers’ sensorimotor, language, cognitive, social and emotional development are at high risk in war conditions (Qouta et al., 2021). Infancy is a highly sensitive period for both developmental risks and protective processes due to intensive neurocognitive, socioemotional and behavioral plasticity (Dawson et al., 2000). This means that infants are open to positive, rewarding and compensatory experiences, such as broad, lively, variable and rhythmically rich maternal singing.

Developmentally informed interventions that enhance resilience and prevent traumatization are urgently needed among children and their families living in war conditions. IDSi has multiple functions in underlying mother–infant emotion sharing, recognition and expression, as well as joy and mutual activation and distress attunement (Ullsten et al., 2018). Interventions with newborns at risk show that music, especially singing, is successful in modulating infants’ affect, pain and distress (Trehub et al., 2015; Ullsten et al., 2017; Virtala & Partanen, 2018). It is thus legitimate to invite mothers to sing with high and rich amplitude and vibration, intensive, pulsative, vivacious, lively and multitudinous rhythm and tone as well as diverse, rich and extensive shapes and movements. High‐quality vocal communication can function as an antidote against the cruel reality of war.

## 5 LIMITATIONS OF THE STUDY

The study can be criticized for using mother‐reported measures of their mental health and infants’ emotional responses, unreliable constructs of infant emotion responses and only a reasonable fit of the tested model for mother‐, infant‐, and war trauma‐related determinants. Clinical interviews would provide a more valid tool to measure mothers’ depression, PTSD, dissociation and somatization symptoms. In addition, observatory methods with expert coding systems are recommended to identify infants’ emotionality and emotion regulation in stressful, challenging and frustrating situations (e.g., the still face paradigm; Tronick, 2003), instead of the vignettes of emotionally significant everyday situations that we used. Parents’ reports about their infants’ characteristics and behaviors can be biased due to their moods and mental health conditions, social expectations and desirability. However, mothers can be real experts in observing early and intimate development because they spend extensive time in dyadic interactions. Pediatric examination is the gold standard of infant health status, and we used it together with mother‐reported infants’ health, indicated by illnesses and disorders. Mothers’ reported war events of destruction were photo‐documented concerning the shelling and bombarding of homes and neighborhoods.

The reasons for the hardly explained variation in the model on mother‐, infant‐, and war trauma‐related factors of IDSi may relate to the fact that maternal singing represents only one element in dyadic communication. Mothers and other caregivers regulate infants’ emotional states through multimodal repetitive and rhythmic vocal, facial, gestural, behavioral, kinesthetic, visual and tactile stimulation (Beebe et al., 2016). A sensitive, mentalizing and emotionally available mother–infant interaction involves multiple microanalytical features and elements to which infants are sensitive from very early on (Slaney & McRoberts, 2003; Tronick & Gianino, 1986). Although singing, as one communication modality by mothers, conveys various temporally coordinated and congruent information, it may not be the core phenomenon of infant development. Therefore, the ecological model of mother‐, infant‐, and society‐related determinants of the quality of acoustic features of IDSi is significant, but other determinants might be more salient.

## 6 CONCLUSIONS

Negative experiences in a mother's life can severely interfere with her favorable musical communication with the infant. Our study confirmed poor quality of IDSi among mothers suffering from mental health problems, caring for infants with poor health status, and exposed to traumatic war events. Characteristic to their unfavorable singing were flat, inarticulate, even and rigid pulsation, beat and tone of vocal amplitude and vibration, unclear, restricted and scant shapes and movements of F0 contours, and low and narrow F0 variability and homogenous tone and scarce, lifeless and monotonous vocal resonance, timbre and rhythm of formants and bandwidths.

Reciprocal, warm, and sensorily rich mother‐infant interaction importantly contributes to good infant mental health. Singing involves multiple acoustic and vocal characteristics that crucially serve infant developmental and mental health functions, such as emotional expression and attunement, cognitive learning, and social exchange. Therefore, both basic research and interventions tailored for infant‐mother shared singing are welcome.

## CONFLICT OF INTEREST STATEMENT

The authors declare no conflicts of interest.

## Supporting information



Supporting Information

## Data Availability

The data serving as a basis of the current study is available at the request of the corresponding author (the first author) in anonymized form. The reason for not being able to provide digital common data is due to the ethical guidelines according to which the participants have been informed about the specific utilization and limited accessibility to the information that they have given to the fieldworkers and researchers.
