# Supplementary material for: The impact of mother's mental health, infant characteristics and war trauma on the acoustic features of infant‐directed singing
Source: Infant Ment Health J. 2025 Aug 3;47(1):e70036. doi: 10.1002/imhj.70036 (PMC12715458; doi:10.1002/imhj.70036)
Supplement: Supplementary file 1 — Supporting Information [file IMHJ-47-0-s001.docx]

SUPPORTIVE INFORMATION Table S1

*Acoustic Features: Definitions and Variables Extracted by OpenSMILE Algorithms of Voice Quality and Measured by Statistical Functions (Variables)*

| Acoustic and prosodic features | Definitions | Variables ^a), d)^ |
| --- | --- | --- |
|  |  |  |
| Fundamental F0 frequency | Acoustic: The lowest periodic cycle component of the acoustic waveform, measured in Hz and reflecting pitch F0 ^b)^  Prosodic: The rate of vibrations of the vocal cords within the larynx, reflects pitch F0 ^b)^ variations of the voice | F0 Mean  F0 Kurtosis  F0 Range (change or difference in F0 = F0 Max – F0 Min)  F0 Skewness  F0 Standard deviation. SD |
| F0 Variability | The Jitter refers to the fast variations in F0 or Difference of Differences of Periods (DDP). The DDP is the average absolute difference between consecutive differences between consecutive periods (divided by the average period) | Jitter DDP Mean  Jitter DDP Kurtosis  Jitter DDP Linre2  Jitter DDP LinreA  Jitter DDP LinreQ  Jitter DDP Quartile 1  Jitter DDP Skewness  Jitter DDP Standard deviation. SD |
| Vocal intensity and energy | Acoustic: The **acoustic intensity** is perceived as the energy and **loudness**of the sound.  Prosodic: Vocal intensity refers to the glottal flow waveform (e.g. peak flow or speed) in terms of lung pressure and phonation threshold pressure.  Energy is assessed through the root-mean-square (RMS) of statistical functions. | RMS Energy Mean  RMS Energy Maximum value (Max)  RMS Energy Range (change or difference in F0=F0 Max – F0 Min)  RMS Energy Skewness  RMS Energy Standard deviation. SD |
| Vocal amplitude and vibration | Acoustic: The **amplitude**of the vibrations (i.e. the size of the oscillations of the vocal folds) affecting the loudness and energy of the voice.  The loudness is indicated through Pulse Code Modulation (PCM) of discrete amplitudes  Prosodic: Energy and loudness refer to the **frequency of vibration** of the vocal folds is perceived as the voice. | PCM Loudness Mean  PCM Loudness IQR differences between 1^st^ and 2^nd^  PCM Loudness IQR differences between 1^st^ and 3^rd^  PCM Loudness IQR differences between 2^nd^ and 3^rd^  PCM Loudness Kurtosis  PCM Loudness Percentage1%  PCM Loudness Percentage99%  PCM Loudness Quartile 1  PCM Loudness Quartile 2  PCM Loudness Quartile 3  PCM Loudness Skewness  PCM Loudness Standard deviation |
| F0 Contours: Shape and movement | Acoustic: The F0 Contour refers to changes over time in the curse of an utterance or to overall shape of voice production in terms of its pitch (F0) variation over time.  The movement in time is indicated by upper and lower envelopes (waves)  Prosodic: The F0 Contour is a realization of the vocal fold oscillation with slowly varying frequencies. Their dynamics are governed by a combination of the length and elasticity of vocal folds, laryngeal muscle tension, and subglottal air pressure.  F0 Envelope: Shape and structures of the movements classified on the basis of their direction and slope. The envelope of the smoothed fundamental frequency contour refers to a rapidly varying signal is a smooth curve outlining its extremes in amplitude [e.g.. flat/unitonal indicating no changes in F0, rising (F0 increases) or falling (F0 decreases)].  . | F0 Contour Beginning Envelope  F0 Contour Envelope Mean  F0 Contour Envelope IQR differences between 1^st^ and 2^nd^  F0 Contour Envelope IQR differences between 1^st^ and 3^rd^  F0 Contour Envelope IQR differences between 2^nd^ and 3^rd^  F0 Contour Envelope Kurtosis  F0 Contour Envelope Percentage1%  F0 Contour Envelope Percentage99  F0 Contour Envelope Quartile 1  F0 Contour Envelope Quartile 2  F0 Contour Envelope Quartile 3  F0 Contour Envelope Skewness  F0 Contour Envelope Standard deviation. SD  F0 Contour Envelope Final  F0 Contour Final Mean  F0 Contour Final IQR differences between 1^st^ and 2^nd^  F0 Contour Final IQR differences between 1^st^ and 3^rd^  F0 Contour Final IQR differences between 2^nd^ and 3^rd^  F0 Contour Final Kurtosis  F0 Contour Final Percentage99  F0 Contour Final Quartile 1  F0 Contour Final Quartile 2  F0 Contour Final Quartile 3  F0 Contour Final Skewness  F0 Contour Final Standard Deviation. SD |
| Formants: Resonance and timbre | Acoustic: A Formant is a concentration of acoustic energy around a particular resonating frequency in the singing wave. Each formant occurs at a different frequency: first (F1) formant 500Hz, second (F2) formant 1500 Hz, and third (F3) formant 2500 Hz. Each formant corresponds to a resonance in the human vocal tract (each bump in the frequency response curve). This study uses the F1 Formant.  Prosodic: Formants can be detected as ridges or peaks in the spectrum of a song signal. Resonances are frequencies where sound waves are amplified by some point of the vocal tract. | The first Formant F1 at 500Hz  Formant F1 Mean  Formant F1 percentage1%  Formant F1 Quartile 2  Formant F1 Quartile 2 – Q - 1  Formant F1 Quartile 3 – Q - 2  Formant F1 Skewness  Formant F1 Standard deviation. SD |
| Rhythmicity through bandwidths | Acoustic: Rhythmicity refers to vocal properties of duration, speed, and intensity, word or vocal stressing, pausing, spectral balances, and regularity in tempo.  Bandwidth of a voice signal is the difference between the higher/upper and lower frequency, measured in Hertz. | Bandwidth Mean  Bandwidth Kurtosis  Bandwidth percentage 1%  Bandwidth Quartile 1  Bandwidth Quartile 2 – Q - 1  Bandwidth Quartile 3  Bandwidth Quartile 3 – Q - 2  Bandwidth Skewness  Bandwidth Standard deviation. SD |
| Vocal tempo and power via attack-time | Acoustic: Attack is the initial impulse required to create oscillation for tempo in utterances. The beginning is defined as change in slope from baseline. Tempo refers to the rise in amplitude (loudness) over time for a given phoneme. To ensure reproducibility and power, the end is defined by maximum amplitude.  Prosodic: The attack-time is analogous to how much an utterance expresses punch and stress Attack-time slope can also change direction. | Attack-time Mean  Attack-time Kurtosis  Attack-time Percentage1%  Attack-time Percentage99  Attack-time Range  Attack-time Skewness  Attack-time Standard Deviation. SD |

Notes ^a)^ Variables extracted by OpenSMILE algorithms of voice quality and indicated by statistical functions.

^b)^ F0 = pitch, referring to the frequency at which vocal chords vibrate in voiced sounds

^c)^ IQR Interquartile Range (IQR) differences between 1^st^ and 2^nd^. 1^st^ and 3^rd^. and 2^nd^ and 3^rd^ waves to indicate shape of the movement and structure

classified based on the direction and slope of movements

^d)^ Variables that are not showing multivariate normality (based on graphical information of the probability-probability, P-P, plots and histograms) were omitted in further analyses. They were from *Fundamental F0 frequency*: F0 Maximum value (Max) and F0 Minimum value (Min); *F0 Variability*: Jitter DDP IQR difference between 1^st^ and 2^nd^ waves, Jitter DDP Percentage99, Jitter DDP IQR difference between 1^st^ and 3^rd^ , Jitter DDP IQR difference between 2^nd^

and 3^rd^ , Jitter DDP Quartile 2, Jitter DDP Quartile 3; *Vocal intensity and energy*: RMS Energy Kurtosis, RMS Energy Minimum value (Min); *Amplitude and vibration*: PCM Loudness Linre2; PCM Loudness LinreQ; *Resonance and timbre via formant*: Formant F1 Kurtosis, Formant F1 Maximum value (Max), Formant F1 Percentage 99, Formant F1 Quartile 1, Formant F1 Quartile 3, Formant F1 Quartile 3; *Rhythmicity via bandwidth*: Bandwidth Maximum value (Max), Bandwidth Minimum value (Min), Bandwidth Percentage 99; Bandwidth Quartile 2; *Vocal tempo and power via attack-time*: Attack-time IQR difference between 1^st^ and 2^nd^ , Attack-time IQR difference between 1^st^ and 3^rd^ , Attack-time IQR difference between 2^nd^ and 3^rd^ , Attack-time Quartile 1, Attack-time Quartile 2, Attack-time Quartile 3.

SUPPORTIVE INFORMATION Table s2 Exploratory Factor Analysis for Acoustic Features of Maternal Infant-directed Singing: Loadings and Communalities

|  | Loadings | | | | | | |
| --- | --- | --- | --- | --- | --- | --- | --- |
| Variables based on algorithms of voice quality ^a)^ | I | II | III | IV | | V | Commu-nality |
| *Fundamental F0 frequency* |  |  |  |  | |  |  |
| F0 Mean ^b)^ | .404 |  |  |  | | -.623 | .667 |
| F0 Kurtosis ^b)^ |  |  |  |  | | .588 | .417 |
| F0 Range ^b)^ |  |  |  | .348 | | -.296 | .326 |
| F0 Skewness ^b)^ | -.364 | . |  |  | | .721 | .699 |
| F0 Standard deviation ^b)^ |  |  |  | .553 | | -.441 | .500 |
| *F0 Variablity* |  |  |  |  | |  |  |
| jitterDDP Mean | .894 |  |  |  | |  | .846 |
| jitterDDP Kurtosis^b)^ | -.586 |  |  | -.374 | |  | .489 |
| jitterDDP Linregc2 | .801 |  |  |  | |  | .678 |
| jitterDDP LinregerrA | .906 |  |  |  | |  | .875 |
| jitterDDP LinregerrQ | .892 |  |  |  | |  | .831 |
| jitterDDP Quartile1 ^b)^ | .418 |  |  |  | | .324 | .357 |
| jitterDDP Skewness | -.710 |  |  |  | |  | .655 |
| jitterDDP Standard deviation | .903 |  |  |  | |  | .864 |
| *Vocal intensity and energy* |  |  |  |  | |  |  |
| pcm_RMSenergy Mean ^b)^ |  | .628 |  |  | | .480 | .756 |
| pcm_RMSenergy Max ^b)^ |  | .433 |  |  | | .537 | .635 |
| pcm_RMSenergy Range ^b)^ |  | .429 |  |  | | .533 | .628 |
| pcm_RMSenergy Skewness ^b)^ |  |  |  |  | | .292 | .142 |
| pcm_RMSenergy Standard deviation ^b)^ |  | .492 |  |  | | .562 | .743 |
| *Vocal amplitude and vibration* |  |  |  |  | |  |  |
| pcm_loudness Mean |  | .965 |  |  | |  | .959 |
| pcm_loudness IQR1-2 |  | .885 |  |  | |  | .831 |
| pcm_loudness IQR1-3 ^b)^ |  | .924 |  |  | |  | .875 |
| pcm_loudness IQR2-3 |  | .894 |  |  | |  | .805 |
| pcm_loudness Kurtosis^b)^ |  | -.610 |  |  | |  | .382 |
| pcm_loudness Percentile1.0 |  | .696 |  |  | |  | .642 |
| pcm_loudness Percentile99.0 |  | .890 |  |  | |  | .812 |
| pcm_loudness Quartile1 |  | .850 |  |  | |  | .791 |
| pcm_loudness Quartile2 ^b)^ | -.252 | .955 |  |  | |  | .935 |
| pcm_loudness Quartile3 |  | .983 |  |  | |  | .982 |
| pcm_loudness Skewness |  | -.676 |  |  | |  | .491 |
| pcm_loudness Standard deviation |  | .920 |  |  | |  | .853 |
| *F0 Contours: shape. and movement* |  | | | |  | |  |
| F0 Contour low envelope |  |  |  |  | |  |  |
| F0finEnvMean ^b)^ | .898 |  |  | .323 | |  | .916 |
| F0finEnv IQR1-2 |  |  |  | .823 | |  | .759 |
| F0finEnv IQR1-3 ^b)^ | -.238 |  |  | .416 | |  | .378 |
| F0finEnv IQR2-3 |  |  |  | .834 | |  | .742 |
| F0finEnv Kurtosis |  |  |  | -.577 | |  | .580 |
| F0finEnv Percentile1.0 ^b)^ | .301 |  |  | .205 | |  | .190 |
| F0finEnv Percentile99.0 |  |  |  | .585 | |  | .724 |
| F0finEnv Quartile1 | .898 |  |  |  | |  | .826 |
| F0finEnv Quartile2 ^b)^ | .932 |  |  | .351 | |  | .899 |
| F0finEnv Quartile3 ^b)^ | .806 |  |  | .373 | |  | .934 |
| F0finEnv Skewness ^b)^ | -.631 |  |  | .414 | |  | .605 |
| F0finEnv Standard deviation ^b)^ | .238 |  |  | .875 | |  | .840 |
| F0 Contour upper envelope |  |  |  |  | |  |  |
| F0final Mean ^b)^ | .896 |  |  | .370 | |  | .941 |
| F0final IQR1-2 |  |  |  | .588 | |  | .415 |
| F0final IQR1-3 ^b)^ | -.265 |  |  | .850 | |  | .805 |
| F0final IQR2-3 |  |  |  | .688 | |  | .574 |
| F0final Kurtosis |  |  |  | -.579 | |  | .500 |
| F0final Percentile99.0 |  |  |  | .597 | |  | .683 |
| F0final Quartile1 | .942 |  |  |  | |  | .888 |
| F0final Quartile2 ^b)^ | .890 |  |  | .351 | |  | .873 |
| F0final Quartile3 ^b)^ | .694 |  |  | .652 | |  | .917 |
| F0final Skewness ^b)^ | -.512 |  | .305 |  | |  | .611 |
| F0final Standard deviation |  |  |  | .925 | |  | .874 |
| *Formants: Voice resonance and timbre* |  |  |  |  | |  |  |
| Formant_0 Mean |  |  | .647 |  | |  | .522 |
| Formant_0_p1 ^b)^ |  |  | .377 |  | |  | .177 |
| Formant_0_q_2 |  |  | .940 |  | |  | .894 |
| Formant_0_q_2_q_1 ^b)^ |  |  | .589 |  | |  | .391 |
| Formant_0 Skewness |  |  | -.825 |  | |  | .699 |
| formant_0 Standard deviation ^b)^ |  |  | .303 |  | |  | .140 |
| *Rhythmicity through Bandwidths* |  |  |  |  | |  |  |
| A_0 Mean ^b)^ |  |  | .966 |  | |  | .936 |
| Bandwidth_0 Kurtosis |  |  | -.819 |  | |  | .679 |
| Bandwidth_0_p1 ^b)^ |  |  | .423 |  | |  | .197 |
| Bandwidth_0_q_2 |  |  | .966 |  | |  | .937 |
| Bandwidth_0_q_2_q_1 |  |  | .976 |  | |  | .959 |
| Bandwidth_0_q_3 |  |  | .975 |  | |  | .953 |
| Bandwidth_0_q_3_q_1 |  |  | .959 |  | |  | .925 |
| Bandwidth_0 Skewness |  |  | -.906 |  | |  | .836 |
| Bandwidth_0 Standard deviation ^b)^ |  |  | .773 |  | |  | .613 |
| *Vocal tempo and power: Attack-time* |  |  |  |  | |  |  |
| Att_time Kurtosis ^b)^ |  |  |  |  | | -.298 | .121 |
| Att_time Range ^b)^ | .597 |  |  |  | |  | .400 |
| Att_time Skewness ^b)^ |  |  |  |  | | -.297 | .125 |
| Att_timeStandard deviation ^b)^ | .692 |  |  |  | |  | .521 |
| Five-factor solution explained 47.89% | 15.44 | 12.05 | 9.44 | 7.13 | | 3.83 |  |

Notes:

^a)^ The headings of factor dimensions are ordered according to the variables in Supplement Table I.

^b)^ The criteria for omitting a variable from further analyses were (1) low communality loading <. 0.50, (2) factor loadings < 0.30, and (3) loadings in two different factors with high loading (Criterion on double loading is that the loadings are >.40 and their difference is < 0.20, Guadagnoli & Velicer, 1988)
